# Microstructural Alterations Analogous to Accelerated Aging of the Cerebral Cortex in Carotid Occlusive Disease

**DOI:** 10.1007/s00062-020-00928-9

**Published:** 2020-07-07

**Authors:** Alexander Seiler, Annemarie Brandhofe, René-Maxime Gracien, Waltraud Pfeilschifter, Elke Hattingen, Ralf Deichmann, Ulrike Nöth, Marlies Wagner

**Affiliations:** 1 Department of Neurology, Goethe University Frankfurt, Schleusenweg 2–16, 60528 Frankfurt, Germany; 2 Brain Imaging Center, Goethe University Frankfurt, Frankfurt, Germany; 3 Institute of Neuroradiology, Goethe University Frankfurt, Frankfurt, Germany

**Keywords:** Cerebral hypoperfusion, Cortical degeneration, Quantitative magnetic resonance imaging, Quantitative (q)T2 mapping, Cortical thickness

## Abstract

**Purpose:**

To investigate cortical thickness and cortical quantitative T2 values as imaging markers of microstructural tissue damage in patients with unilateral high-grade internal carotid artery occlusive disease (ICAOD).

**Methods:**

A total of 22 patients with ≥70% stenosis (mean age 64.8 years) and 20 older healthy control subjects (mean age 70.8 years) underwent structural magnetic resonance imaging (MRI) and high-resolution quantitative (q)T2 mapping. Generalized linear mixed models (GLMM) controlling for age and white matter lesion volume were employed to investigate the effect of ICAOD on imaging parameters of cortical microstructural integrity in multivariate analyses.

**Results:**

There was a significant main effect (*p* < 0.05) of the group (patients/controls) on both cortical thickness and cortical qT2 values with cortical thinning and increased cortical qT2 in patients compared to controls, irrespective of the hemisphere. The presence of upstream carotid stenosis had a significant main effect on cortical qT2 values (*p* = 0.01) leading to increased qT2 in the poststenotic hemisphere, which was not found for cortical thickness. The GLMM showed that in general cortical thickness was decreased and cortical qT2 values were increased with increasing age (*p* < 0.05).

**Conclusion:**

Unilateral high-grade carotid occlusive disease is associated with widespread cortical thinning and prolongation of cortical qT2, presumably reflecting hypoperfusion-related microstructural cortical damage similar to accelerated aging of the cerebral cortex. Cortical thinning and increase of cortical qT2 seem to reflect different aspects and different pathophysiological states of cortical degeneration. Quantitative T2 mapping might be a sensitive imaging biomarker for early cortical microstructural damage.

## Introduction

Atherosclerotic large vessel stenosis is one of the leading causes for ischemic stroke worldwide [1]. Apart from being a known stroke risk factor [2], internal carotid artery occlusive disease (ICAOD) is associated with cognitive impairment [3, 4], even in otherwise neurologically asymptomatic patients without evident brain lesions due to ischemic stroke or white matter changes on conventional structural magnetic resonance imaging (MRI) [3]. The presence of high-grade carotid stenosis has been associated with decreases of cerebral gray matter volume and progressive atrophy of the cerebral cortex [5], suggesting an influence of ICAOD on cortical microstructural integrity, which may mediate impairment of cognitive functions. Cortical thickness reflects the total content of neurons, the cellular composition and the cytoarchitectonic organization of a given cortical region [6] and therefore MRI-based estimation of cortical thickness is frequently used to investigate the integrity of the cerebral cortex on the microstructural level. A previous study demonstrated a significant relationship between unilateral carotid stenosis and widespread bilateral cortical thinning [7]. Other studies found an association between hemodynamic impairment and focal cortical thinning [8–11], suggesting a direct detrimental effect of carotid occlusive disease on cortical microstructure; however, a recent study could not replicate this finding and found no influence of carotid stenosis on ipsilateral or contralateral cortical thickness in a multivariate analysis [12]. Thus, given the complex nature of cortical microstructural alterations associated with ICAOD and the different possible pathophysiological states of cortical tissue damage, other imaging modalities might be useful to reveal the direct hemodynamic effect of ICAOD on cortical microstructure that might go beyond volume decrease as a relatively advanced stage of cortical damage. Quantitative (q)MRI with qT2 mapping is sensitive to pathological tissue alterations, such as increase of tissue water content due to extracellular and intracellular edema, gliosis, demyelination and axonal damage, which typically lead to a prolongation of qT2 values [13, 14]. Given these underlying pathophysiological mechanisms, increases of cortical qT2 in general should be associated with cortical thinning but may be assessable before reduction of cortical thickness occurs. The aim of this study was to investigate the cortical microstructural integrity in patients with unilateral ICAOD by means of cortical thickness estimation and qT2 mapping as complementary imaging techniques. We hypothesized that sensitivities of both methods to pathological tissue changes may be different due to a reflection of different pathophysiological states of the same microstructural cortical pathology. Consequently, supplementing the estimation of cortical thickness with qT2 mapping might have the potential to improve the detectability of early pathological impairment of the cortical microstructure. While previous studies mainly used cortical thickness and thus a volume-related measure for investigating alterations of cortical microstructure in ICAOD, this study aimed to provide insights into possible microstructural tissue properties underlying and preceding cortical volume reduction. A group of control subjects was used for comparison to assess potential bilateral changes of cortical microstructure in ICAOD.

## Material and Methods

### ICAOD Patients and Control Subjects

A total of 22 patients (2 female) with Doppler/ultrasound or computed tomography (CT) angiography evidence of a unilateral symptomatic or asymptomatic stenosis (≥70%) or occlusion of the extracranial or intracranial ICA were included in this single center study between January 2015 and December 2016. Patients were recruited from the neurovascular outpatient clinic and the neurovascular ward of our neurological department. Similar sample sizes were used in previous studies described in the literature, which investigated cortical thickness [11, 15] and T2 mapping [16] in patients with cerebral large artery steno-occlusive disease. Therefore, we expected that with this patient collective and control group, having a similar size, significant differences in cortical thickness and cortical qT2 values would be detectable between patients and controls. In addition, we performed a power analysis with the intended statistical model and the entire sample size for our study (for further details, see “Statistical Analysis”) using the R software (“simr” package, version 1.0.5, R foundation, Vienna, Austria). In this power analysis, the simulations resulted in a reasonable statistical power of 73.3%, and 79.2% for detecting an effect of one standard deviation for cortical qT2 values and half a standard deviation for cortical thickness according to the results of previous studies from the literature [15, 16]. The larger effect for qT2 values was postulated because of the projected optimized surface-based analysis of cortical qT2 values with sampling of qT2 maps to the cortical ribbon (see “Further Image Postprocessing and Analysis”). Grading of stenosis was performed according to the North American Symptomatic Carotid Endarterectomy Trial (NASCET) criteria. Extracranial carotid occlusion was determined and distinguished from near occlusion based on established Doppler/ultrasound criteria [17]: (1) the lack of a flow signal in the ICA on duplex and color Doppler flow sonography with increased low-flow sensitivity (low pulse repetition frequency, high color enhancement) at complete visibility of the entire vessel structure in the B‑mode [18], (2) activated collateral vessels and (3) reduced flow velocity in the ipsilateral common carotid artery with increased pulsatility. Intracranial carotid occlusion was verified by digital subtraction angiography. Exclusion criteria were the presence of ischemic lesions involving the cerebral cortex and the presence of an extracranial or intracranial stenosis of ≥50% of another artery supplying the brain. Patients with a symptomatic vessel pathology were eligible for inclusion in the study if they only had smaller subcortical ischemic lesions (<1 cm^3^ infarct volume) in the territory of the affected artery. Activation of primary collateral pathways as an indicator of poststenotic hemodynamic impairment in ICAOD patients was assessed by transcranial Doppler/ultrasound, evaluating the presence of retrograde flow in the ophthalmic artery (OA) and the A1 segment of the anterior cerebral artery (ACA A1) ipsilateral to the vessel pathology. In addition, the peak systolic velocity (PSV) of the middle cerebral arteries (MCA) was assessed by transcranial Doppler/ultrasound as a further indicator of hemodynamic impairment. From a physiological point of view, the PSV of the poststenotic/postocclusive MCA depends on (1) the reduction of perfusion pressure across the vessel pathology (in case of a stenosis), (2) the magnitude and efficacy of collateral flow to the poststenotic MCA via the collateral pathways of the circle of Willis and thus to the affected vascular territory, and (3) the vascular resistance of the distal vasculature in the affected hemisphere. Thus, it reflects the severity of the hemodynamic impairment and the degree of adaptation of the cerebral vasculature. For comparison of imaging parameters reflecting cortical microstructural integrity, we recruited a group (*n* = 20, 11 female) of healthy control subjects. Control subjects were enrolled based on the following criteria: (1) age between 50 and 80 years, (2) no history of neurological or psychiatric disease, (3) no history of smoking, diabetes, hyperlipidemia or untreated arterial hypertension. Criteria (2) and (3) were ensured using medical records and standardized questionnaires. Sex was not expected to have a relevant influence on the hemispheric mean values of cortical thickness and cortical qT2, according to previous studies [19, 20]. The study was approved by the local ethics committee of the Goethe University Frankfurt (Faculty of Medicine) and written informed consent was obtained from all subjects before inclusion in the study.

### MRI Protocol

The MRI data were acquired on a 3T whole-body scanner (Magnetom Trio; Siemens, Erlangen, Germany) using the body coil of the scanner for radiofrequency transmission and an 8‑channel phased array head coil for signal reception. The MR imaging examination included quantitative T2 mapping as well as conventional T1 and T2-weighted sequences.

Anatomic imaging for tissue segmentation was based on a T1-weighted magnetization prepared rapid acquisition of gradient echoes (MPRAGE) sequence with the following imaging parameters: TR/TE/TI = 1900/3.04/900 ms, FOV = 256 × 256 × 192 mm^3^, whole-brain coverage, isotropic spatial resolution = 1 mm, bandwidth = 170 Hz/pixel, excitation angle = 9°, parallel imaging with reduction factor of 2, duration = 4 min 28 s.

Quantitative T2 mapping was based on a fast spin-echo sequence with an echo-train length of 11 echoes per excitation, an echo spacing of 17.1 ms, and the following imaging parameters: 35 axial slices with 2‑mm slice thickness, no interslice gap, TR = 7000 ms, band width = 100 Hz/pixel, 180° refocusing pulses, matrix size = 192 × 144 (readout × phase encoding), FOV = 240 × 180 mm^2^, and in-plane resolution = 1.25 × 1.25 mm^2^. For quantitative T2-mapping, 5 datasets were acquired with different TE values (17, 86, 103, 120, 188 ms), keeping all other acquisition parameters constant. The total duration was 8 min 55 s.

### Calculation of qT2 maps

To reduce the influence of subject motion, a target data set was created by calculating the sum of squares average of the five T2-weighted data sets acquired at different TE. Subsequently, the individual source data sets were coregistered to this target. Quantitative T2 maps were obtained pixel-wise from the five coregistered data sets by mono-exponentially fitting the dependence of the T2-weighted signal amplitude on TE.

### Further Image Postprocessing and Analysis

Individual T1-weighted anatomic MPRAGE images were processed with the Freesurfer software (version 6.0.1, Athinoula A. Martinos Center for Biomedical Imaging, Charlestown, MA, USA) using the recon-all stream with standard parameters for tissue segmentation and surface-based reconstruction of the cortical ribbon [21, 22], which enables the indirect calculation of the local cortical thickness [23].

**Fig. 1:**
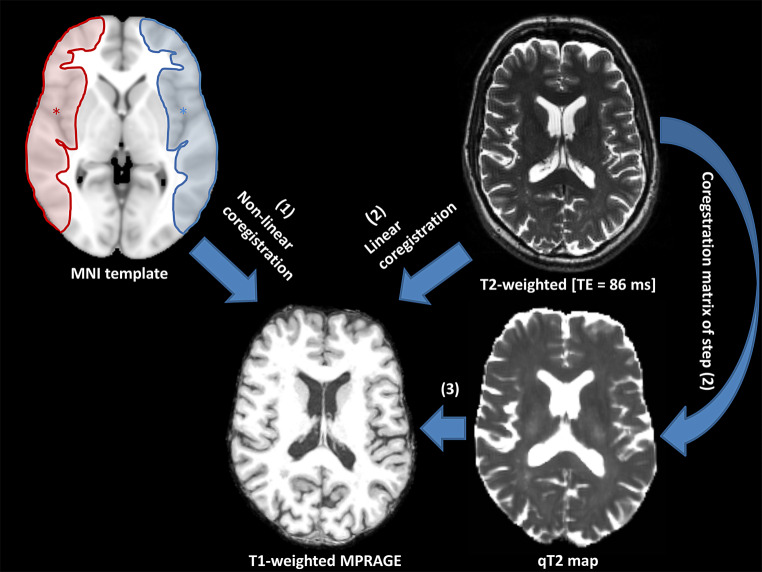
Illustration of regions of interest (ROI) placement and coregistration procedures. ROIs covering the cortical portion of the MCA territory were created in MNI standard space according to an atlas of the cerebral vascular territories and were then non-linearly coregistered (1) to each individual T1-weighted anatomic image in Freesurfer image space. Steps (2) and (3) involve linear coregistration. *MPRAGE* magnetization prepared rapid acquisition of gradient echos, *MNI* Montreal Neurological Institute, *TE* echo time, *q* quantitative

Image coregistration was performed with tools provided in the Functional Magnetic Resonance Imaging of the Brain (FMRIB) software library (FSL, version 5.0.7) [24]. Each subject’s qT2 map was linearly coregistered to the respective skull-stripped T1-weighted image in the Freesurfer image space (T1.mgz) in the following way: the second T2-weighted image (TE = 86 ms), which provides sufficient anatomic contrast for coregistration, was linearly coregistered to the T1-weighted image (Fig. 1). Subsequently, the resulting coregistration matrix was applied to the qT2 map for coregistration of the quantitative data to the anatomic image (Fig. 1).

For spatially coherent analysis of cortical thickness and cortical qT2 values, the latter were mapped onto the individual cortical surface. In order to account for the poorer spatial resolution of qT2 compared to the MPRAGE images and to minimize partial volume effects to the adjacent cerebrospinal fluid and subcortical white matter, qT2 maps were sampled at the midcortical depth (interval of the cortical depth fraction 0.4–0.6), thereby excluding 40% of the cortical tissue fraction near the subcortical white matter and 40% of the cortical tissue fraction near the outer pial surface [25].

**Fig. 2:**
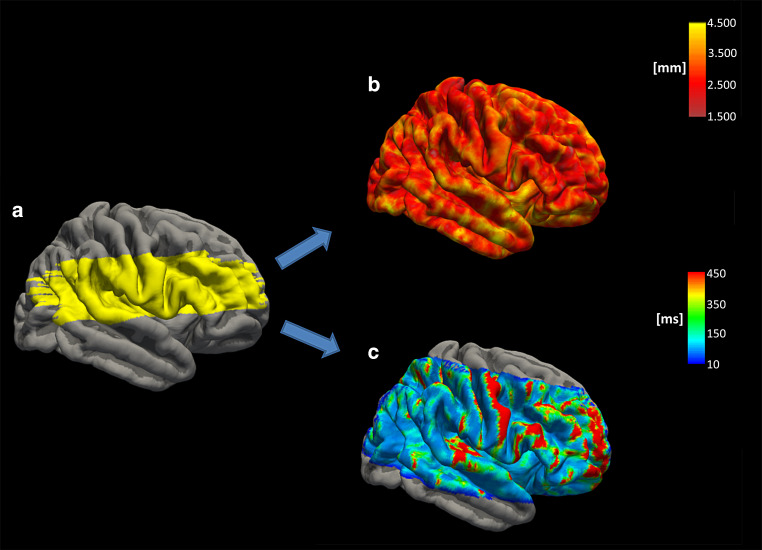
Surface-based analysis of cortical thickness and cortical qT2 values. After mapping onto the cortical surface (**a**), ROIs of the cortical part of the MCA territory were used to extract mean cortical thickness (**b**) and cortical qT2 values (**c**). The ROI for the cortical part of the right MCA territory derived from MNI standard space as well as cortical thickness and cortical qT2 values of one representative control subject are shown on the pial surface of the right hemisphere of the average control subject (fsaverage) created by the Freesurfer software. Cortical qT2 values were sampled at the midcortical depth to minimize partial volume effects from subcortical white matter and cerebrospinal fluid. Note that qT2 mapping was not acquired with full brain coverage. *mm* millimeters, *ms* milliseconds

For the investigation of the hypothesized effect of hemodynamically relevant ICAOD on cortical microstructure in the dependent vascular territory and the homologous contralateral area, a region of interest (ROI)-based approach was used. The ROIs covering the cortical proportion of the middle cerebral artery (MCA) territory on each side were created in Montreal Neurological Institute (MNI 152) standard space (1 mm isotropic resolution) according to the vascular territories of the human brain described by Tatu et al. ([26]; Fig. 1, upper left image). The ROIs were non-linearly coregistered from the MNI standard T1 standard template to the individual patient-specific T1-weighted image using the following approach: the T1-weighted MNI standard template was coregistered to the T1-weighted anatomic image with a rigid transformation followed by an affine transformation. The affine transformation was used as an initialization for non-linear coregistration. Finally, the warp field coefficients obtained from the non-linear coregistration were applied to coregister the ROIs from MNI standard space to each individual T1-weighted anatomic image (Fig. 1). The proportion of each ROI which intersected with the cortical tissue fraction as identified by reconstruction of the cortical ribbon was mapped onto the cortical surface (Fig. 2a) and mean values for cortical thickness and cortical qT2 were extracted from each ROI (Fig. 2c,d). Previous studies investigating cortical thickness in patients with cerebrovascular disease showed a strong association between white matter lesions (WML) of vascular origin and reduced cortical thickness [27, 28]. Given the high prevalence of WML in patients with carotid occlusive disease, we considered the WML volume as a potential influencing factor regarding the investigated imaging parameters. Therefore, WML for each subject were manually segmented (including earlier subcortical infarcts) for each subject on the individual second T2-weighted image (TE = 86 ms) by two experienced neuroradiological raters in consensus using the MRIcron software (Chris Rorden, Columbia, SC, USA; www.mricro.com). Here, standard consensus criteria were applied to identify WML of vascular origin [29] and T1-weighted anatomic images were taken into consideration to ensure accuracy, e.g. in differentiating WML from prominent perivascular spaces [29]. A binary WML mask was created by manual delineation of the lesions for each subject. The individual volume of the segmented WML was determined by calculating the volume of the lesion mask.

### Statistical Analysis

Statistical analyses were performed using SPSS Statistics 26 (IBM Corp. Armonk, NY, USA). For the investigation of significant effects of independent variables on cortical thickness and cortical qT2 values, two separate generalized linear mixed models (GLMM) were fitted, in which the two hemispheres were included as repeated measures. The independent variables included in each model consisted of group (patient/control), hemisphere (right/left), and the presence of upstream carotid stenosis (yes/no). Age and WML volume (cm^3^) were included as covariates. Baseline variables between groups were compared with the Mann-Whitney U-test. The Wilcoxon-signed rank test was used to compare cortical thickness and cortical qT2 values between the hemisphere ipsilateral to the stenosis and the contralateral hemisphere in ICAOD patients. For assessment of a significant relationship between cortical thickness and cortical qT2 values Spearman’s rank correlation was used. Statistical significance was set to *p* < 0.05.

## Results

Mean age of ICAOD patients was lower compared to healthy controls (64.8 ± 11.2 years vs. 70.8 ± 7.3 years, *p* = 0.075). Out of 22 patients in the ICA stenosis group, 19 (86%) had an extracranial ICA pathology, while the intracranial ICA was affected in 3 (14%) patients. Of the patients, 7 (31.8%) had an ICA occlusion (6 extracranial and 1 intracranial), while the remaining 15 patients (68.2%) had a high-grade ICA stenosis (range of degree of stenosis: 70–95%) and 1 patient showed a tandem pathology with a proximal extracranial stenosis of 70% and an intracranial stenosis of 80%. The right ICA was affected in 15 patients (68.2%), while the other 7 patients (31.8%) had a left-sided vessel pathology, 7 (31.8%) of the ICAOD patients had an asymptomatic vessel pathology and 15 (68.2%) had suffered a minor subcortical stroke or a transient ischemic attack in the territory of the affected artery and 16 patients (72.7%) in the ICAOD group had activated collaterals as assessed by transcranial Doppler/ultrasound (8 patients with retrograde flow in the OA or ACA A1, 8 patients with retrograde flow in both OA and ACA A1). The PSV of the MCA ipsilateral to the ICA pathology was significantly reduced in comparison to the contralateral side (69± 28.1 cm/s vs. 97.4± 27.32 cm/s, *p* = 0.002). There was no significant difference between ICAOD patients and healthy controls regarding WML volume (3.22± 2.56 cm^3^ vs. 3.03± 3.78 cm^3^, *p* = 0.302). Reconstruction of the cortical surface and thus computation of cortical thickness failed in one healthy control subject for technical reasons, while qT2 maps could be analyzed for all subjects included in the study.

**Fig. 3:**
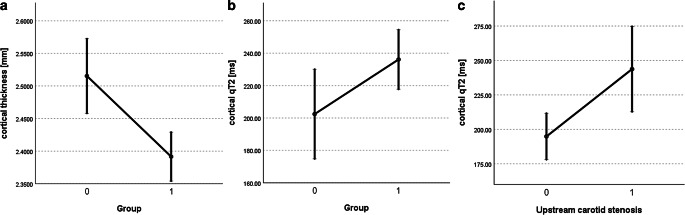
Mean values and standard errors for cortical thickness (**a**) and cortical qT2 values (**b**,**c**) depending on the independent variables “Group” and “Upstream carotid stenosis” in the generalized linear mixed model. While only “Group” showed a significant main effect on cortical thickness (**a**), both “Group” (**b**) and the presence of upstream carotid stenosis (**c**) had a significant main effect on cortical qT2 values. *1* denotes ICAOD patients (**a**,**b**) and presence of upstream carotid stenosis (**c**), while *0* denotes control subjects (**a**,**b**) and absence of upstream carotid stenosis (**c**). *mm* millimeters, *ms* milliseconds

The GLMM analyses showed a significant main effect of the group on both cortical thickness (*p* = 0.034) and cortical qT2 values (*p* = 0.023) with reduced cortical thickness and increased cortical qT2 in patients compared to controls (Tables 1 and 2; Fig. 3a,b). The presence of upstream carotid stenosis had a significant main effect on cortical qT2 (*p* = 0.01) leading to an increase of qT2 values (Table 2; Fig. 3c). For cortical thickness as the dependent variable, no significant effect of the presence of stenosis (*p* = 0.411) was found (Table 1). In ICAOD patients, cortical qT2 values ipsilateral to the stenosis/occlusion were significantly elevated by comparison to the contralateral hemisphere (264.87 ± 90.13 ms vs. 212.68 ± 54.71 ms, *p* = 0.003), while cortical thickness ipsilateral to the vessel pathology was smaller compared to the contralateral side without reaching statistical significance (2.3808 ± 0.1613 mm vs. 2.4053 ± 0.1389 mm, *p* = 0.445).

**Table 1:** Main effects on cortical thickness in the first generalized linear mixed model

Independent variable	Coefficient	*p*-value	Coefficient 95% CI
Group = patients (patients < controls)	−0.492	*0.034*	–0.945 to –0.038
Hemisphere = right (right < left)	0.001	0.968	–0.054 to 0.056
Age (covariate)	−0.008	*0.01*	–0.013 to –0.002
WML volume (covariate)	−0.01	0.08	–0.021 to 0.001
Upstream carotid stenosis = yes (yes < no)	−0.031	0.411	–0.107 to 0.044

*WML* white matter lesion, *CI* confidence interval

**Table 2:** Main effects on cortical qT2 values in the second generalized linear mixed model

Independent variable	Coefficient	*p*-value	Coefficient 95% CI
Group = patients (patients > controls)	258.018	*0.023*	36.271 to 479.764
Hemisphere = right (right > left)	3.383	0.805	–23.788 to 30.554
Age (covariate)	2.916	*0.04*	0.136 to 5.695
WML volume (covariate)	3.321	0.219	–2.012 to 8.654
Upstream carotid stenosis = yes (yes > no)	48.911	*0.01*	12.091 to 85.731

*WML* white matter lesion, *CI* confidence interval

The WML volume did not have a significant main effect on cortical thickness (*p* = 0.08) or cortical qT2 values (*p* = 0.219) in the multivariate analyses (Tables 1 and 2); however, there was a significant main effect of age on both cortical thickness and cortical qT2 (*p* = 0.01 and *p* = 0.04) with decreasing thickness and increasing qT2 values at increasing age (Tables 1 and 2). The hemisphere (right/left) as an independent variable had no significant main effect on cortical thickness (*p* = 0.968) or cortical qT2 values (*p* = 0.805) (Tables 1 and 2). By between hemisphere comparisons (right vs. left) in healthy control subjects no significant differences were found regarding cortical thickness and cortical qT2 values (*p* = 0.136 and *p* = 0.681).

After stratification of the ICAOD patients into two groups according to the status of collateral activation (with/without collateral activation), no significant differences were found for cortical thickness and cortical qT2 values in the hemisphere ipsilateral and contralateral to the stenosis/occlusion between the groups. For cortical qT2 values ipsilateral to the vessel pathology, there was a strong trend towards significance suggesting higher cortical qT2 in patients with activated collaterals (288.74 ± 89.07 ms vs. 201.21 ± 60.29 ms, *p* = 0.059). The ICAOD patients with activated collaterals had significantly lower PSV values in the poststenotic/postocclusive MCA as compared to patients without collateral activation (58.93 ± 16.63 cm/s vs. 99.2 ± 35.4 cm/s, *p* = 0.018) and a significantly more pronounced relative decrease of PSV in the poststenotic/postocclusive MCA in comparison to the contralateral MCA (−37.7 ± 17.55% vs. +4.68 ± 21.76%, *p* = 0.006). The PSV obtained from the poststenotic/postocclusive MCA showed a significant negative correlation with the ipsilateral cortical qT2 values (r = −0.491, *p* = 0.028), which was not found with ipsilateral cortical thickness (r = −0.062, *p* = 0.794). Symptomatic (*n* = 15) and asymptomatic (*n* = 7) patients did not show significant differences in terms of cortical thickness and cortical qT2 values, neither in the contralateral hemisphere nor in the ipsilateral hemisphere to the vessel pathology (*p*-values between 0.091 and 0.368).

**Fig. 4:**
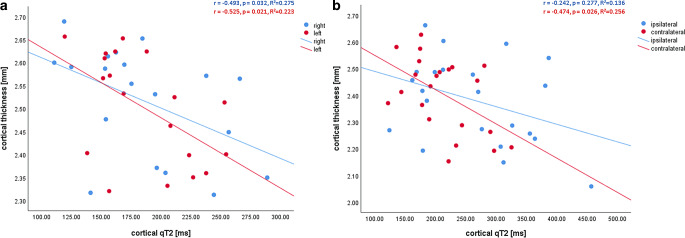
Scatterplots illustrating the relationship between cortical qT2 values and cortical thickness in healthy control subjects (**a**) and in ICAOD patients (**b**) after stratification according to the respective hemispheres (right/left for control subjects, ipsilateral/contralateral to the stenosis for ICAOD patients). In ICAOD patients, in the hemisphere ipsilateral to the vessel pathology, there is an increase of cortical qT2 values which is not accompanied by a concurrent decrease of cortical thickness, leading to a flatter slope of the fitted linear regression line (**b**). *mm* millimeters, *ms* milliseconds

In healthy control subjects cortical thickness and cortical qT2 values were significantly negatively correlated in both hemispheres (right hemisphere: r = −0.525, *p* = 0.021, left hemisphere: r = −0.493, *p* = 0.032) (Fig. 4a). This significant negative correlation was also found in ICAOD patients in the hemisphere contralateral to the stenosis (r = −0.474, *p* = 0.026, Fig. 4c), but not in the hemisphere ipsilateral to the stenosis (r = −0.242, *p* = 0.277, Fig. 4b). Correlations were corrected for multiple comparisons by performing false discovery rate (FDR) correction (corrected level of significance: q < 0.038).

## Discussion

This study investigated the imaging parameters cortical thickness and qT2 as plausible markers of potential damage to the cortical microstructure in patients with unilateral high-grade ICAOD in comparison to an older cohort of healthy control subjects. Independent of the hemisphere, ICAOD patients showed decreased cortical thickness and increased cortical qT2 values compared to control subjects, indicating a global and not only a unilateral effect of ICAOD on cortical microstructure (Tables 1 and 2, Fig. 3a,b). Interestingly, the presence of upstream carotid pathology had a significant main effect on cortical qT2 values resulting in significantly increased cortical qT2 values in the MCA territory ipsilateral to the vessel pathology as compared to the contralateral side (Table 2; Fig. 3c). This effect was not found in the analysis on cortical thickness as the dependent variable and cortical thickness did not differ between the two hemispheres in ICAOD patients (Table 1).

Estimation of cortical thickness is commonly mainly used as a surrogate of cortical neuronal density [9] while cortical qT2 mapping is sensitive to pathological processes like intracellular and extracellular edema, gliotic tissue conversion and damage of myelin and axonal structures [13, 14, 16]. Both reduction of cortical thickness and prolongation of cortical qT2 ipsilateral to a high-grade carotid stenosis/occlusion have been found in previous studies, which suggested an association of these cortical tissue alterations with hemodynamic impairment [8–11, 16]. In this study, significant bilateral cortical thinning and increase of cortical qT2 values were found in ICAOD patients, involving both the ipsilateral dependent and the corresponding contralateral vascular territory. In view of the demonstrated association of decreased cortical thickness and increased qT2 with hemodynamic impairment in patients with a high-grade occlusive carotid pathology [8–11, 16] and the widespread character of bilateral changes in imaging parameters, compromised blood flow may be assumed as the pathophysiological mechanism driving changes of cortical thickness and cortical qT2. Indeed, reduced parenchymal cerebral blood flow (CBF) has been associated with progressive decrease of cortical gray matter volume in patients with high-grade ICAOD [5]. Chronic cerebral hypoperfusion and the resulting impairment of brain energy metabolism in patients with unilateral ICAOD can also affect the contralateral hemisphere through activation of collateral pathways [30–32], which was found in the majority of our patients, resulting in a relative shortage of blood and substrates [30, 33, 34]. Activation of collateral pathways in unilateral high-grade ICAOD is generally associated with hemodynamic compromise and reduced cerebral vascular reactivity which predominantly affect the hemisphere ipsilateral to the steno-occlusive vessel pathology but also the contralateral hemisphere. This can be explained by the fact that collaterals are usually not fully compensating and thus in some respect reflect the severity of hemodynamic impairment [32, 35]. In this study, consistent with this notion, ICAOD patients with collateral activation exhibited lower ipsilateral MCA flow velocity and a significantly more pronounced relative decrease of the ipsilateral MCA flow velocity compared to the contralateral side. Concordantly, there was at least a strong trend towards higher cortical qT2 values ipsilateral to the ICA pathology in patients with collateral recruitment. These findings suggest an increase of cortical qT2 values related to collateral activation which is mediated by hemodynamic impairment as the actual cause. Furthermore, it has been shown that impairment of cerebral vasoreactivity as an indicator of exhausted autoregulatory capacity, which is associated with atrophy of cortical gray matter [36], occurs in both hemispheres in patients with unilateral high-grade ICAOD [32, 37, 38]. Consequently, it is plausible that reduced CBF as a result of a drop in perfusion pressure distal to the vessel pathology is responsible for the bilateral pathological alterations of cortical microstructure. Critical hypoperfusion leads to an impaired delivery of oxygen and energy substrates in the cerebral microcirculation which may induce a neurodegenerative process [5, 39, 40]. Since the cortical gray matter is particularly prone to hypoperfusion due to its high demand regarding oxygen and glucose supply [41], relative bilateral hypoperfusion plausibly explains the pathological alterations of cortical microstructure as the causative mechanism.

In addition to the global cortical thinning and increase of cortical qT2 values found in ICAOD patients, there is an independent effect of carotid stenosis on the ipsilateral cortex causing an extensive prolongation of cortical qT2 (Table 2; Fig. 3c) compared to the contralateral side. This finding is best explained by the unilaterally pronounced chronic hemodynamic compromise [42] caused by a high-grade carotid stenosis and the subsequent harmful effect on the cortical microstructure [16]. Strongly supporting this hypothesis, there was a significant reduction of the MCA flow velocity ipsilateral to the ICA pathology as compared to the contralateral side and the poststenotic/postocclusive PSV was significantly negatively correlated with ipsilateral cortical qT2 values, indicating increasing cortical qT2 with compromised antegrade flow to the affected vascular territory. While in some studies ipsilateral cortical thinning was detected and could be directly linked to cerebral blood flow impairment [8–11], a recent study on patients with unilateral ICA stenosis failed to demonstrate this effect [12]. This might be partly due to the fact that the extent of cortical thickness reduction associated with cerebral hypoperfusion is generally small and thus not necessarily reproducible in smaller patient collectives with only moderate hypoperfusion. Furthermore, cortical thinning is commonly assumed to reflect mainly neuronal loss and involution of the neuropil [9] and thus an early stage of cortical atrophy. Therefore, pathological alterations of cortical microstructure that precede changes of the cortical volume fraction might not be captured by estimation of cortical thickness. This interpretation of reduced cortical thickness as a consequence of neuroglial involution and thus of a degenerative process may apply only to adults, where both aging and pathological conditions are associated with a decline of cortical thickness [27]. During adolescence, the decrease of cortical thickness associated with increasing age occurs due to cortical restructuring with elimination of inefficient synaptic connections and proliferation of myelin, leading to a refinement of neural circuits [43–46]. Therefore, cortical thinning during adolescence reflects cortical maturation and is not indicative of a degenerative process; however, in this study on older subjects, the association of reduced cortical thickness with increased cortical qT2 values indicates a degenerative process. Generally, increase of cortical qT2 values and cortical thinning are closely linked (Fig. 4a,b) and presumably represent the same underlying pathological development. These pathological tissue changes represent cortical microstructural damage as a degenerative process ultimately leading to cortical atrophy. Considering the fact that cortical thinning represents volume reduction (even though subtle), while qT2 mapping is sensitive to pathological alterations which may not necessarily go along with changes of the cortical volume fraction at an early stage, these two imaging parameters presumably reflect both different aspects and different pathophysiological states of cortical microstructural pathology. An independent effect of upstream carotid stenosis was not found concerning cortical thickness (Table 1) and there was no significant difference in cortical thickness between the ipsilateral and the contralateral hemisphere in ICAOD patients. Thus, in the cortex ipsilateral to the stenosis, there seems to be an overproportional increase of cortical qT2 which is not accompanied by a concurrent reduction of cortical thickness (Fig. 4b), for which reason the significant negative relationship between cortical thickness and cortical qT2 values is not maintained (Fig. 4b). This suggests pathological processes such as gliosis, microstructural enlargement of the extracellular compartment due to neuronal involution, loss of myelin and axonal damage that increase quantitative transverse relaxation time and precede reduced cortical thickness. Compared to estimation of cortical thickness, qT2 mapping might be a more sensitive imaging marker for early hypoperfusion-related tissue damage.

As expected [12, 14], there was a significant general effect of age on both cortical thickness and cortical qT2 values with decreased cortical thickness and increased cortical qT2 values at higher age (Tables 1 and 2). Thus, given the younger mean age of ICAOD patients compared to the healthy control subjects, chronic hemodynamic impairment seems to trigger a degenerative process which may equate to anticipated aging of the cerebral cortex and provide a link to cognitive impairment in carotid occlusive disease. It is also known that CBF generally decreases with age [47–49]; however, since it is not entirely clear whether reduced cerebral perfusion is a cause or a consequence of cortical atrophy at all in normal brain aging [50, 51], and whether hypoperfusion-related and merely age-related alterations of cortical microstructure show different properties on the histological level, this has to be interpreted with caution.

Subcortical infarcts are known to cause focal cortical thinning in connected ipsilateral and contralateral cortical areas [52, 53]. In addition, previous studies investigating cortical thickness in subjects with white matter hyperintensities suggested both global and region-specific associations between cortical thickness and WML lesion volume [27, 28]. Yet, in our study WML lesion volume did not significantly affect cortical thickness and cortical qT2 values in multivariate analyses (Tables 1 and 2). Furthermore, WML lesion volumes did not differ significantly between patients and control subjects and therefore group differences regarding imaging parameters in any case would not be explainable by differences in WML lesion volumes. This finding supports the assumption that the association between high-grade ICAOD and pathological alterations of cortical microstructure is directly mediated by a restriction of blood flow through the hemodynamically relevant large vessel pathology [16] and not by a widespread distal microvascular pathology. Thus, in contrast to what has been suggested earlier [7, 54], ICAOD in this respect might not only be an indicator of generalized atherosclerosis affecting the cortical microstructure by direct involvement of the cerebral microcirculation. Although the macrovascular blood flow velocity as measured with Doppler/ultrasound is related to the regional blood flow of the more distal vasculature, it does not allow assessment of the hemodynamic situation on the tissue level in affected brain parenchyma. Therefore, a combination of cortical thickness and qT2 mapping with arterial spin labeling (ASL) as a technique for the absolute quantification of CBF would be of great interest for future studies and permit an evaluation of cortical thinning and cortical qT2 increase related to hypoperfusion of the respective cortical region. Furthermore, the combination with territorial vessel-encoded ASL would allow the investigation of collateral flow patterns [32, 55, 56], the magnitude of collateral blood flow, and thus the relationship between cortical qT2 values and collateral supply.

In this study, estimation of cortical thickness and cortical qT2 mapping were used as two complementary imaging parameters, presumably reflecting different aspects of the same tissue pathology, with a ROI-based approach. The multivariate analyses allowed separate investigations of both a general and a side-specific effect of unilateral ICAOD on cortical thickness and cortical qT2 values, while considering and controlling for other influencing factors. Furthermore, the linear mixed models employed in this study accounted for the fact that a subject’s two hemispheres should not be regarded as statistically independent from each other, while enabling the inclusion of the hemispheres as repeated measures. Most of the ICAOD patients showed WML and thus did not have lesion-free brains. Although the actual causative link between the presence of ICAOD and the magnitude of cerebral WML is still unknown [57, 58], the general association between ICAOD and bilateral WML is well-documented [59, 60]. Therefore, the patient selection in this study allowed investigation of a somewhat representative patient collective with respect to the general cross-section of ICAOD patients.

## Limitations

This study has several limitations. First of all, the overall sample sizes of both groups are relatively small which may limit the detection of significant differences in cortical thickness as the magnitude of cortical thickness changes is generally small. It should be noted that the acquisition of quantitative MRI data may require relatively long measurement times and that multi-echo sequences in general are susceptible to subject motion, which can be problematic in patients with cerebrovascular disease. Thus, the eligibility of patients for an imaging study may be limited by these factors, especially for patients with acute or subacute neurological symptoms or other relevant comorbidities. Furthermore, our patient collective was heterogeneous in terms of the clinical course of the vessel pathology and patients and control subjects were not perfectly matched in all aspects. Although we controlled for subcortical ischemic lesions in our analysis, we cannot completely exclude certain concealed differences between symptomatic and asymptomatic patients, potentially influencing our results. Yet, our analysis was not powered to investigate an impact of the clinical course on the imaging parameters or an association between imaging markers of cortical microstructural integrity and clinical outcome. Since this was not a longitudinal study, we are not able to comment further on the significant negative relationship between cortical thickness and cortical qT2 values. Even though it is plausible that pathological changes, which cause a prolongation of transverse relaxation time antecede measurable cortical thinning, it cannot be ruled out that cortical thickness itself may have an influence on cortical qT2; however, this would not question the results of this study with respect to the proven effects of carotid stenosis on cortical thickness and cortical qT2 values. Finally, we did not systematically assess clinical features, such as cognitive performance and therefore we cannot relate our results to clinical findings.

## Conclusion

In conclusion, our results demonstrate global cortical thinning and increased cortical qT2 values in patients with unilateral high-grade ICAOD even when compared to an older cohort of control subjects, despite the general influence of age on both cortical thickness and cortical qT2 in multivariate analyses. This finding suggests a harmful effect of chronic cerebral hypoperfusion on cortical microstructure in both hemispheres, which may be similar to accelerated aging processes of the cerebral cortex. Additionally, there is an independent effect of high-grade carotid stenosis on the ipsilateral cortex, which causes an extensive prolongation of cortical qT2 and is presumably mediated by unilaterally pronounced chronic hemodynamic impairment. Increases of cortical qT2 seem to precede cortical atrophy with reduced cortical thickness due to the sensitivity of qT2 mapping to pathological microstructural alterations that take place before cortical volume reduction occurs. Therefore, qT2 might be suitable to depict early cortical microstructural damage and may be helpful to identify patients who could still benefit from revascularization. Further research is necessary to explore the clinical relevance of cortical qT2 changes in patients with carotid occlusive disease, especially with respect to potential cognitive impairment.
